# Novel Epstein-Barr virus-like particles incorporating gH/gL-EBNA1 or gB-LMP2 induce high neutralizing antibody titers and EBV-specific T-cell responses in immunized mice

**DOI:** 10.18632/oncotarget.13770

**Published:** 2016-12-01

**Authors:** Elizabeth M. Perez, Joslyn Foley, Timelia Tison, Rute Silva, Javier Gordon Ogembo

**Affiliations:** ^1^ Department of Medicine, University of Massachusetts Medical School, MA, USA; ^2^ Department of Experimental Therapeutics, Beckman Research Institute of City of Hope, Duarte, CA, USA

**Keywords:** Epstein-Barr virus, viral glycoproteins, viral latency proteins, virus-like particles, vaccine

## Abstract

Previous Epstein-Barr virus (EBV) prophylactic vaccines based on the major surface glycoprotein gp350/220 as an immunogen have failed to block viral infection in humans, suggesting a need to target other viral envelope glycoproteins. In this study, we reasoned that incorporating gH/gL or gB, critical glycoproteins for viral fusion and entry, on the surface of a virus-like particle (VLP) would be more immunogenic than gp350/220 for generating effective neutralizing antibodies to prevent viral infection of both epithelial and B cell lines. To boost the humoral response and trigger cell-mediated immunity, EBV nuclear antigen 1 (EBNA1) and latent membrane protein 2 (LMP2), intracellular latency proteins expressed in all EBV-infected cells, were also included as critical components of the polyvalent EBV VLP. gH/gL-EBNA1 and gB-LMP2 VLPs were efficiently produced in Chinese hamster ovary cells, an FDA-approved vehicle for mass-production of biologics. Immunization with gH/gL-EBNA1 and gB-LMP2 VLPs without adjuvant generated both high neutralizing antibody titers *in vitro* and EBV-specific T-cell responses in BALB/c mice. These data demonstrate that EBV glycoprotein(s)-based VLPs have excellent immunogenicity, and represent a potentially safe vaccine that will be invaluable not only in preventing EBV infection, but importantly, in preventing and treating the 200,000 cases of EBV-associated cancers that occur globally every year.

## INTRODUCTION

Infection with Epstein-Barr virus (EBV), an oncogenic gamma-herpesvirus, is associated with the development of lymphoproliferative disorders and over 200,000 cancer cases worldwide every year [1, 2]. Preclinical and clinical studies of EBV candidate vaccines have demonstrated that boosting the immune response against EBV can limit viral infection and protect both animals and humans from developing EBV-related diseases [3–6]. Yet EBV vaccine development has met several challenges, including: the potential oncogenic effects of introducing EBV DNA/RNA using virions; limited production and release of the virus particles to be used as vaccine; and propagation of many vaccine candidates in transformed human cell lines, limiting the ability to meet the stringent FDA safety guidelines. For example, recent phase I clinical trials of recombinant modified vaccinia Ankara (MVA) vector encoding EBV nuclear antigen 1 (EBNA1) and latent membrane protein 2 (LMP2) as a vaccine candidate elicited a robust EBV-specific CD4^+^ and CD8^+^ T-cell response in humans [6–8]. However, the use of MVA to deliver these two important EBV antigens, known for their oncogenic potential, may pose major health risks, such as integrating oncogenic DNA in an undesired location of the host genome, and potentially causing unchecked cell growth, particularly in immunosuppressed individuals [9, 10]. There is also a risk of vaccine tolerance, since the proteins are constantly produced *in vivo.*

Another major limitation of vaccines currently in pre-clinical and clinical trials is that none of the vaccines has created sterile immunity (i.e., complete blockage of viral infection), perhaps because most of the strategies only target one arm of the immune system, either humoral (e.g., major immunodominant EBV surface glycoprotein gp350/220, prophylactic vaccine) [2, 11] or cell-mediated (e.g., MVA-EBNA1-LMP2, therapeutic vaccine). Even in cases where both arms of the immune system have been targeted in a single vaccine, such as with the use of EBV DNA packaging mutants [12, 13], the vaccine candidates were met with safety concerns.

Virus-like particles (VLPs) overcome many of these challenges. VLPs lack the viral genome and typically assemble from one or more viral structural proteins, forming repetitive arrays that resemble a natural virus [14]. Importantly, VLPs are known for strong immunogenicity and safety in all populations, regardless of immune status [15]. The recent success of subunit-based VLP vaccines targeting other oncogenic viruses (e.g., human papillomavirus and hepatitis B virus [16, 17]), suggests a similar strategy could be safe and effective for EBV. Our Newcastle disease virus-like particle (ND VLP) platform consists of expression vectors encoding recombinant EBV genes of interest and key NDV structural proteins, which are co-transfected into mammalian host cells for assembly and production of Epstein-Barr VLPs (EB VLPs) containing the target proteins [18–20]. Notably, ND VLPs provide a platform for the inclusion of multiple select viral surface glycoproteins and intracellular T-cell antigens to generate a polyvalent vaccine, which could stimulate both humoral and cell-mediated immune responses for effective EBV prevention and control.

EBV membrane glycoproteins, which are critical for viral entry and infection of host epithelial and B cells [21, 22], provide attractive humoral immune response targets for inclusion in a prophylactic EBV vaccine. In EBV infection, the attachment surface glycoprotein gp350/220 makes first contact with host B-cell receptors CD21 and CD35 [23–26], and subsequently triggers endocytosis of the virions [27]. Fusion between the viral envelope and host cellular membrane receptors is the next required step in the entry of all human herpesviruses [21]; for EBV, the necessary viral envelope glycoproteins include gH and gL [28–30], gp42 [31], and gB [32]. Importantly, co-expression of gH and gL is required for gL transport to the cell surface, and formation of a stable cell surface complex of gH/gL [33]. Together, these glycoproteins mediate EBV entry into both epithelial and B cells, and thus they facilitate infection and confer host cell specificity. Fusion with epithelial cells is facilitated by both gH/gL and gB [32–34], while B-cell tropism is defined by gH/gL and gp42 [35].

Although neutralizing antibodies (nAbs) directed to EBV envelope glycoproteins are present in humans, prevent neonatal infection, and are generated in response to EBV infection in humans, or to immunization with virus glycoproteins in humans and other animals [3, 36, 37], persistent EBV infection and evidence of limited immune selection of viral antigenic variants indicate that *in vivo* neutralization of EBV infection is suboptimal. In support of these observations, in four independent phase I/II clinical trials, vaccination with vector constructs expressing gp350/220 or with the purified recombinant non-splicing variant, gp350, soluble protein did not prevent infection, although acute infectious mononucleosis (AIM) was reduced in young adults [3, 4, 38, 39]. Importantly, primary B-cells can be infected with recombinant EBV lacking gp350/220, suggesting that additional viral ligands mediating EBV infection in the absence of gp350/220 may exist [40]. These observations indicate that using gp350/220 as the only immunogen to target viral neutralization is not optimal and may account for the variable success of this protein in EBV vaccine development [3–5, 11, 38]. This evidence drove our use of other critical EBV glycoproteins (gH/gL and gB) as alternative vaccine targets for generating an effective antibody response in immunized mice and in our development of a polyvalent vaccine.

Much evidence establishes EBV intracellular latency proteins EBNA1 and LMP2 as attractive targets for stimulation of a cell-mediated immune response in a therapeutic EBV vaccine candidate [6–8, 41–46]. Both are expressed in all EBV-infected cells, including EBV-related tumors [1]. EBNA1 and LMP2-specific CD4^+^ and CD8^+^ T cells are frequently detected in EBV-infected individuals [43, 47, 48], and both T-cell subsets can be effective in controlling growth of EBV-immortalized epithelial or B cells [8, 39, 44, 45]. Furthermore, immunosuppression of EBV-positive individuals typically leads to EBV-associated lymphomas and post-transplant lymphoproliferative disorders (PTLDs) [49]. Adoptive transfer of EBV-specific T cells can induce remission in transplant patients [50, 51], suggesting that T cell-mediated responses are effective in controlling persistent EBV infection.

In this study, we provide evidence that subunit VLPs can incoporate EBV surface glycoproteins, package intracellular antigens, and that the VLPs can be stably produced in the Chinese hamster ovary (CHO) cell line. The polyvalent EB VLPs can stimulate both humoral and T cell-mediated immune responses in wild-type BALB/c mice. To our knowledge, these combinations have not yet been tested in pre-clinical or clinical trials as part of a prophylactic and therapeutic EBV vaccine candidate.

## RESULTS

### VLPs that incorporate gp350/220-F on the surface and package eGFP-NP intracellularly are stably produced in CHO cells

VLPs have typically been used to express only surface glycoproteins, for stimulation of the humoral immune response [14]. In addition, current production of most VLPs requires repeated transient transfections of multiple plasmid components. This process is laborious, expensive, and results in varying yields of VLPs. As a proof of concept, we generated a novel polyvalent EBV vaccine that can package both surface glycoproteins and intracellular proteins, for stimulation of both humoral and cell-mediated immune responses, and is stably expressed in CHO cells following a single transfection.

**Figure 1:**
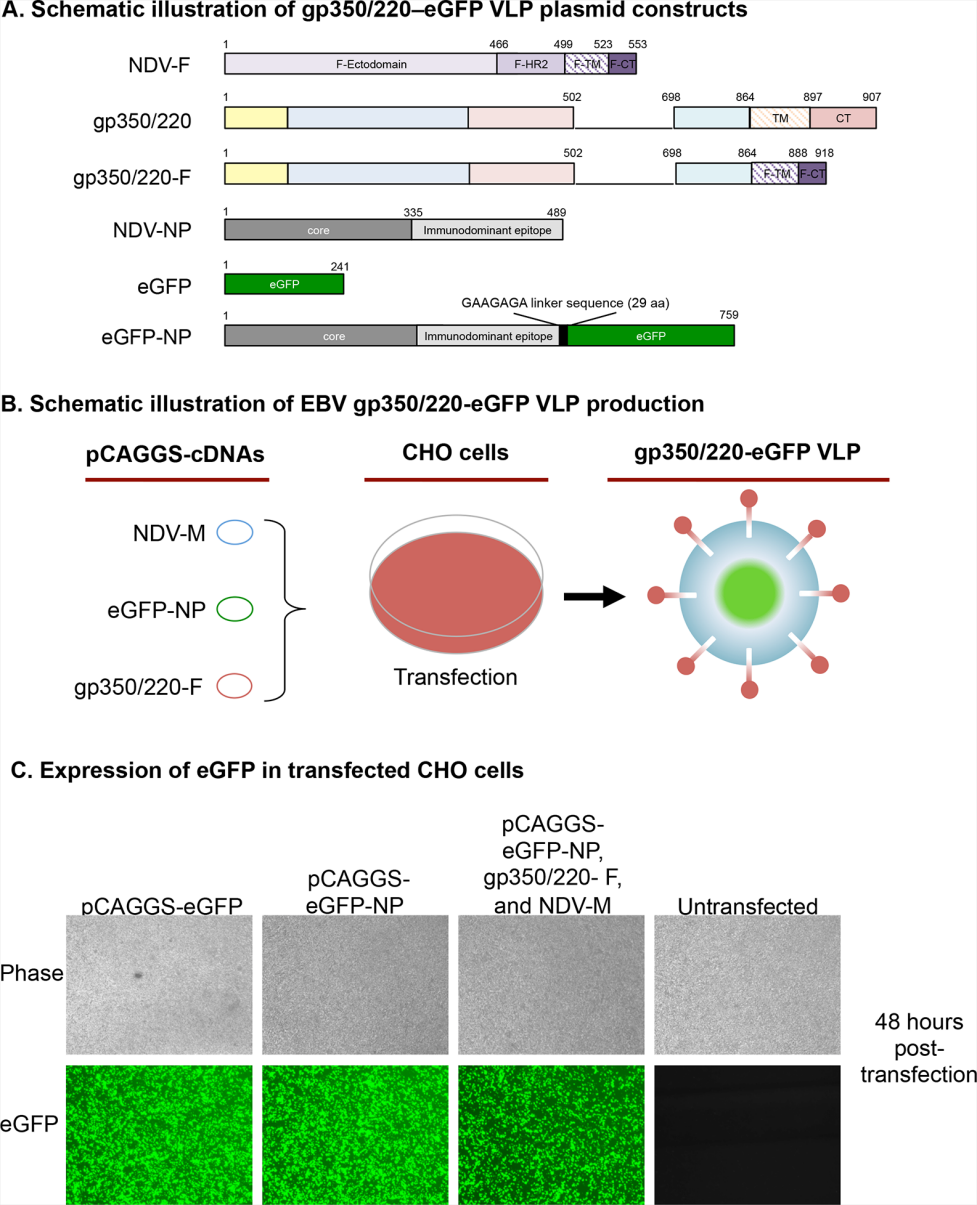

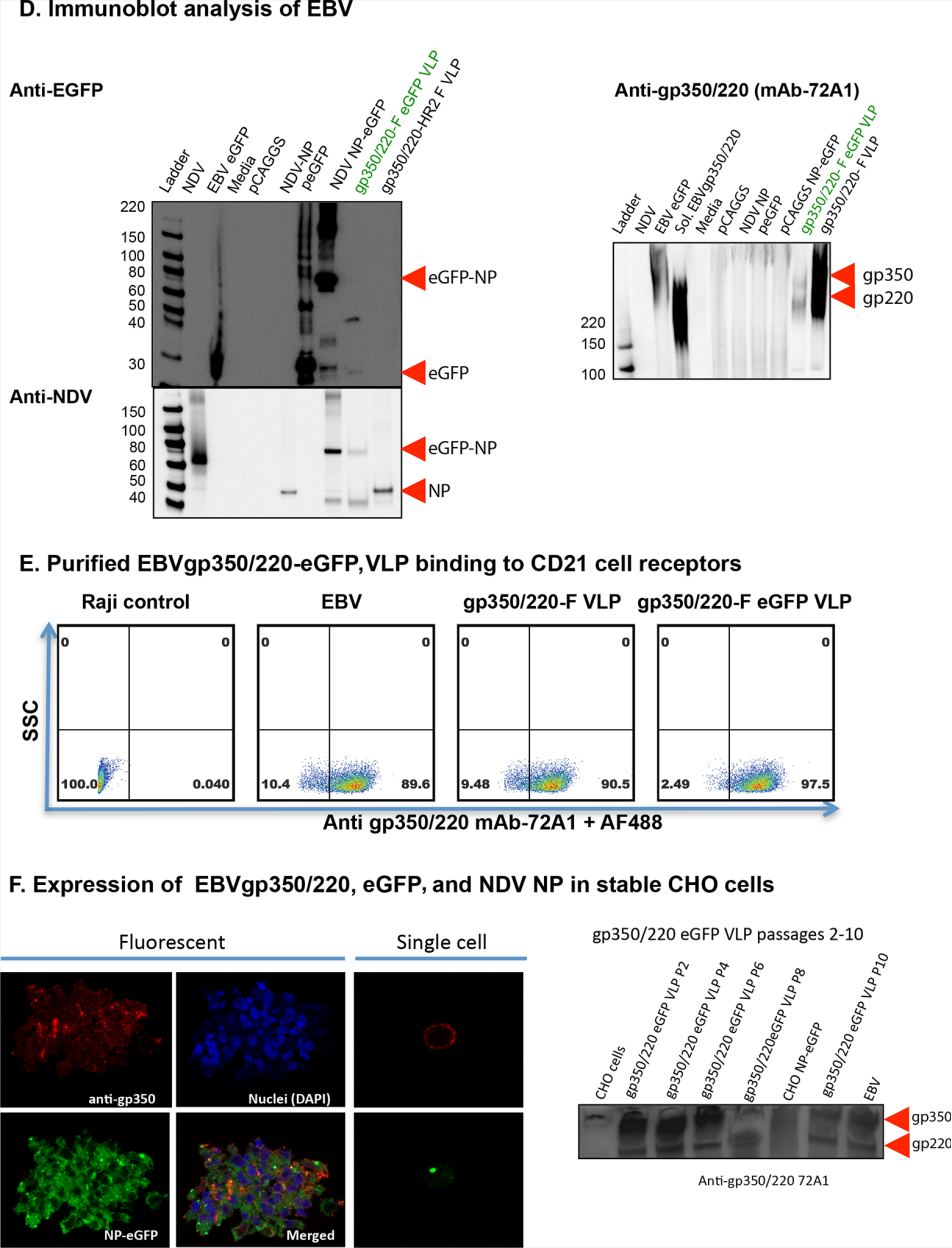
Assembly and characterization of gp350/220-eGFP-NP VLPs **A.** Schematic illustrations (not to scale) of gp350/220-eGFP VLP plasmid constructs depicting the full length NDV-F, gp350/220 (wild type), and the gp350/220-F (chimeric) as well as the NDV-NP, eGFP, and fusion of eGFP-NP cDNAs. **B.** A schematic workflow for the transfection of CHO cells with three required VLP components, NDV M, eGFP-NP, and gp350/220-F, resulting in gp350/220-eGFP VLPs. **C.** Fluorescence microscopy comparing eGFP expression in CHO cells transfected with pCAGGS-eGFP, pCAGGS-eGFP-NP, and the three VLP components in comparison to untransfected CHO cells, showing that all three conditions result in eGFP expression at nearly equal levels. **D.** VLP immunoblots confirming eGFP (top panel to the left), NDV (bottom panel to the left), and gp350/220 (panel to the right) expression. The anti-eGFP blot detected eGFP protein in cells transfected with various eGFP plasmids, EBV eGFP, as well as in VLPs containing eGFP. The anti-NDV (a gift of Dr. T. Morrison, University of Massachusetts Medical School), detects expression of the eGFP-NP fusion protein as well as NP. Finally, the anti-gp350/220 blot shows expression of both gp350 and 220 in both VLPs. **E.** To confirm that the VLPs bound to the surface receptor CD21, purified EBV and gp350/220 VLPs were incubated with Raji cells (expressing CD21), stained with anti-gp350/220, and FACs analysis was performed to see if the VLPs would bind to the receptor and how well they bound in comparison to eGFP-EBV. At least ~90% of the cells fluoresced green when either virus or VLP was bound. **F.** Confocal microcopy confirmed that CHO cells transfected with the three VLP components stably expressed both gp350/220 (on the surface of the cells, red color) and NP-eGFP (predominantly in the nucleus, green color). DAPI was used to stain the nucleus. Immunoblot of purified VLPs also detected expression of gp350/220 after passaging CHO 10 times, indicating that the gp350/220-eGFP VLPs can be stably produced in CHO cells.

To demonstrate that both surface glycoprotein(s) and intracellular proteins can be packaged in a VLP, we generated plasmids encoding genes to be incorporated as part of the EB VLP, surface glycoprotein (gp350/220) and enhanced green fluorescent protein (eGFP), a marker for intracellular expression. First, we synthesized chimeric sequences of EBV gp350/220 surface glycoprotein fused to the NDV fusion (F) protein transmembrane (TM)/cytoplasmic (CT) domains (gp350/220-F; Figure 1A, top panel) and eGFP fused to NDV nucleocapsid protein (NP) (eGFP-NP; Figure 1A, bottom panel). To assemble and produce gp350/220-eGFP VLPs, equal amounts of pCAGGS-gp350/220-F and pCAGGS-eGFP-NP chimeras were co-transfected into CHO cells, together with pCAGGS-NDV matrix (M) protein (Figure 1B). pCI-puro was included in the transfection for the selection of stable cells.

To confirm successful transfection and intracellular expression of chimeric protein eGFP-NP, we used fluorescence microscopy to visualize expression of eGFP in CHO cells 48 hours post-transfection. Over 80% of cells transfected with pCAGGS plasmids expressing eGFP alone (control); eGFP-NP; or eGFP-NP, gp350/220-F, and M (i.e., gp350/220-eGFP VLPs) expressed eGFP-NP protein (Figure 1C). VLP-producing CHO cells were expanded and VLPs were purified as described [18, 20]. To demonstrate that CHO cells assembled and released gp350/220-eGFP VLPs, we confirmed expression of component proteins using immunoblot of purified gp350/220-eGFP VLPs and relevant controls (Figure 1D). Anti-eGFP detected eGFP and/or eGFP-NP chimeric proteins in lysates from B95-8-eGFP cells, in CHO cells transfected with pCAGGS-eGFP or pCAGGS-eGFP-NP (positive controls), as well as in purified gp350/220-eGFP VLPs (top panel to the left). Media alone, NDV lysate, CHO cells transfected with empty pCAGGS vector (negative controls), CHO cells transfected with pCAGGS-NP, and purified gp350/220 VLPs did not express eGFP. Anti-NDV, which recognizes NDV NP, detected NP and/or eGFP-NP in CHO cells transfected with pCAGGS-NP or pCAGGS-eGFP-NP, and in NDV lysate (positive controls), as well as in purified gp350/220-eGFP and gp350/220 VLPs (which contain NP as a VLP constituent). Lysates from B95-8-eGFP cells, media alone, and CHO cells transfected with empty pCAGGS vector (negative controls), or CHO cells transfected with pCAGGS-eGFP did not express NP (bottom panel to the right). Anti-gp350/220 monoclonal antibody (mAb; 72A1) detected gp350/220 and/or gp350/220-F chimeric proteins in lysates from B95-8-eGFP cells as well as soluble gp350/220 (positive controls), and in purified gp350/220-eGFP and gp350/220 VLPs. NDV lysate, media alone, and CHO cells transfected with empty pCAGGS vector (negative controls), CHO cells transfected with pCAGGS-NP, pCAGGS-eGFP, or pCAGGS-eGFP-NP did not express gp350/220 (right panel). Thus, the purified gp350/220-eGFP VLPs contained the appropriate gp350/220-F surface glycoprotein and eGFP-NP intracellular components. To confirm that the gp350/220-F chimeric protein expressed on the VLPs was functional, we tested for binding of purified VLPs to the gp350/220 cognate receptor, CD21. We incubated purified VLPs with Raji cells, known to express CD21, and used flow cytometry with anti-gp350/220 (72A1) to detect bound VLPs (Figure 1E). Untreated Raji cells (negative control) demonstrated no gp350/220 binding. Raji cells incubated with purified gp350/220 or gp350/220-eGFP VLPs showed gp350/220 bound to 90.5% and 97.5% of cells, respectively, similar to purified B95-8-eGFP (positive control).

To generate a colony of stable VLP-producing cells, CHO cells transfected with pCAGGS-gp350/220-F, pCAGGS-eGFP-NP, pCAGGS-M and pCI-puro were selected with 10 μg/ml of puromycin 48 hours post-transfection. Five colonies of stable cells were randomly selected, expanded, and sorted by flow cytometry using eGFP and anti-gp350/220 mAb 72A1. Sorted stable cells were expanded and VLPs were purified as above. Confocal microscopy analysis of the stable cells for eGFP expression, and immunoblot analysis of VLPs purified from stable cells, revealed that stable cells produced VLPs equivalent to those produced in the initial transfection, up to passage 10, without any remarkable loss in quantity of gp350/220 in purified VLPs (Figure 1F). These results confirm that the EBV VLP platform can be used to package both EBV intracellular and surface glycoproteins, and that VLPs can be stably produced in CHO cells following a single transfection step.

### Construction, purification, and characterization of gp350/220-EBNA1, gH/gL-EBNA1, or gB-LMP2 VLPs

Although the major EBV envelope glycoprotein gp350/220 has been proposed as a potential vaccine antigen, attempts to develop a potent gp350/220-based vaccine in the past four decades have had limited success [2, 11, 52]. We thus hypothesized that an alternate envelope glycoprotein, such as the complex gH/gL or gB, which are essential for EBV fusion and entry, would be a critical component for an effective antibody-mediated prophylactic vaccine. EBV-related malignancies such as Burkitt lymphoma, Hodgkin's disease, and nasopharyngeal carcinoma typically express EBV nuclear antigen 1 (EBNA1) and varying levels of latent membrane protein 2 (LMP2) [1]. Thus, we reasoned that incorporating EBNA1 or LMP2 into gp350/220, gH/gL or gB VLPs, respectively, would improve immunogenicity by targeting the cell-mediated arm of the immune system, in addition to the humoral arm targeted by the surface glycoprotein components.

**Figure 2:**
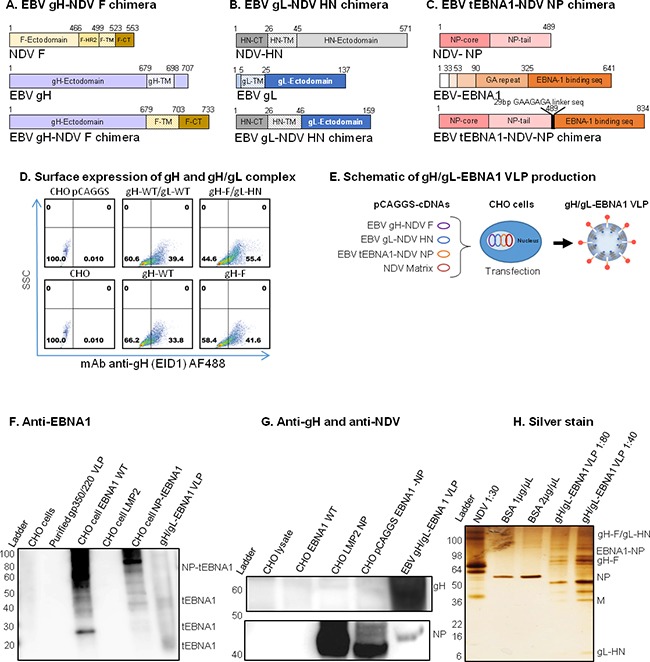
Construction, assembly, and characterization of gH/gL-EBNA1 VLPs Schematic illustrations (not to scale) of **A.** EBV gH-NDV F, **B.** EBV gL-NDV HN, and **C.** EBV tEBNA1-NDV NP chimera plasmids. **D.** pCAGGS-cDNAs of the two chimeras and NDV Matrix were co-transfected into CHO cells for VLP production as illustrated. **E.** Flow cytometry for surface expression of gH and gH/gL on 106 CHO cells transfected with 1μg of (clockwise from top left): empty pCAGGS vector (neg. control); pCAGGS-gH-full-length (WT) + gL-WT (pos. control); pCAGGS-gH-F + gL-HN chimeras; gH-F chimera; gH-WT; no transfection (CHO). SSC, side scatter; Anti-gH/gL mAb E1D1 binds gH alone or gH/gL complex (a gift of L. Hutt-Fletcher Louisiana State University, Shreveport). **F.** Purified gH/gL-EBNA1 VLPs and transfected cells were lysed and analyzed by immunoblot with mAb anti-DNA binding domain EBNA1 (a gift of F. Grässer, Institut für Virologie, Germany) to detect EBNA1. **G.** Sera from mice immunized with UV-KSHV was used to detect gH. Polyclonal anti-NDV was used to detect NP proteins (a gift of Dr. T. Morrison, University of Massachusetts Medical School, Worcester, Polyclonal anti-NDV MA), and anti-mouse sera from mice immunized with UV-inactivated EBV, as indicated (top panel). Anti-EBNA1: un-transfected CHO cells, purified gp350/220 VLP, and CHO cells transfected with LMP2 were neg. controls; CHO cells transfected with EBNA1-WT or NP-tEBNA1 chimera were pos. controls. Anti-NDV: un-transfected CHO cells and CHO cells transfected with EBNA1-WT were neg. controls; NDV was pos. control. **H.** Purity of EBV gH/gL-EBNA1 VLPs was determined by Silver stain, and VLP component proteins of predicted sizes were detected as indicated; NDV was pos. control, BSA was used for quantification.

To demonstrate the efficacy of packaging both viral surface glycoproteins (gp350/220 or gH/gL) and an intracellular latency protein (EBNA1) inside a VLP, we used a similar strategy as above to generate both gp350/220-EBNA1 (Supplementary Figure S1) and gH/gL-EBNA1 VLPs (Figure 2). To construct the gH/gL-EBNA1 VLP, we synthesized chimeric sequences of: EBV gH ectodomain fused to the NDV F TM/CT domains (gH-F, Figure 2A); EBV gL ectodomain fused to NDV hemagglutinin-neuraminidase (HN) protein TM/CT domains (gL-HN, Figure 2B); and EBV truncated (t) EBNA1 fused to full-length NDV NP (tEBNA1-NP, Figure 2C). Given that endogenously expressed full-length EBNA1 is protected from cytotoxic T lymphocyte recognition, making its recognition and presentation to MHC class I a challenge, we reasoned that to make an immunogenic vaccine, a truncation of EBNA1 would be necessary. In this truncation, the Gly-Ala region, known to impair presentation of *cis*-linked sequences (amino acids 1-325) to MHC class I is deleted. The fragments were individually cloned into pCAGGS and cDNA fidelity was verified by sequencing.

To demonstrate that chimeric gH-F and gL-HN form gH/gL complexes at the surface of the transfected cells, similar to full length gH/gL or gH alone, we transfected CHO cells with the relevant pCAGGS plasmids. At 48 hours post-transfection, cells were scraped, stained with anti-gH (E1D1) (Figure 2D) or anti-gH (CL59) (data not shown), and analyzed by flow cytometry. gH and/or gH/gL surface proteins were detected on CHO cells co-transfected with full-length gH and gL or with chimeric gH-F and gL-HN, as well as CHO cells transfected with full-length gH only or the gH-F chimera. Untransfected CHO cells, or CHO cells transfected with empty pCAGGS plasmid, served as negative controls. Detection of gL alone was not performed due to lack of commercial antibody.

To assemble and produce the gH/gL-EBNA1 VLPs, equal amounts of cDNAs (8 μg/T-175 flask) of pCAGGS-gH-F, pCAGGS-gL-HN, and pCAGGS-tEBNA1-NP chimeras were co-transfected into CHO cells, together with pCAGGS-M (Figure 2E). VLPs were purified as previously described [18, 20]. To confirm the production of gH/gL, tEBNA1, M, and NP VLP components, purified gH/gL-EBNA1 VLPs were analyzed for protein composition and molecular weight using immunoblot (Figure 2F–2G) and silver stain (Figure 2H). Anti-EBNA1 directed to the DNA binding domain detected tEBNA1 and/or tEBNA1-NP chimeric protein in CHO cells transfected with pCAGGS-EBNA1 full-length or pCAGGS-tEBNA1-NP (positive controls), as well as purified gH/gL-EBNA1 VLPs, but not in untransfected CHO cells (negative control), purified gp350/220 VLPs, or CHO cells transfected with pCAGGS-LMP2. Anti-NDV detected NP and/or tEBNA1-NP chimeric protein in purified gH/gL and gH/gL-EBNA1 VLPs, as well as NDV lysate (positive control), but not in untransfected CHO cells (negative control), or CHO cells transfected with pCAGGS-EBNA1. As an effective antibody against gH/gL has not been optimized for immunoblotting, silver stain was used to detect VLP component proteins as well as to assess VLP purity. NDV lysate was used as a positive control; BSA was used for quantification of protein. Bands for gH/gL-EBNA1 VLP component proteins of predicted sizes were detected for chimeric tEBNA1-NP (109 Kda), gH/gL (130 Kda), gH-F (85 Kda), NDV NP (53 Kda), NDV M (40 Kda), and gL-HN (25 Kda). Even though E1D1 was recently shown to detect the gH/gL complex in immunoblot [53], in our hands, this antibody did not detect gH/gL in purified EBV, in cells transfected with gH/gL, or in VLPs in both non-reducing and reducing buffers. Further review of the literature did not identify any other previously published article detecting gH/gL with E1D1 in immunoblot, a fact confirmed by Dr. L Hutt-Fletcher (Louisiana State University Health Sciences Center, Shreveport, LA).

**Figure 3:**
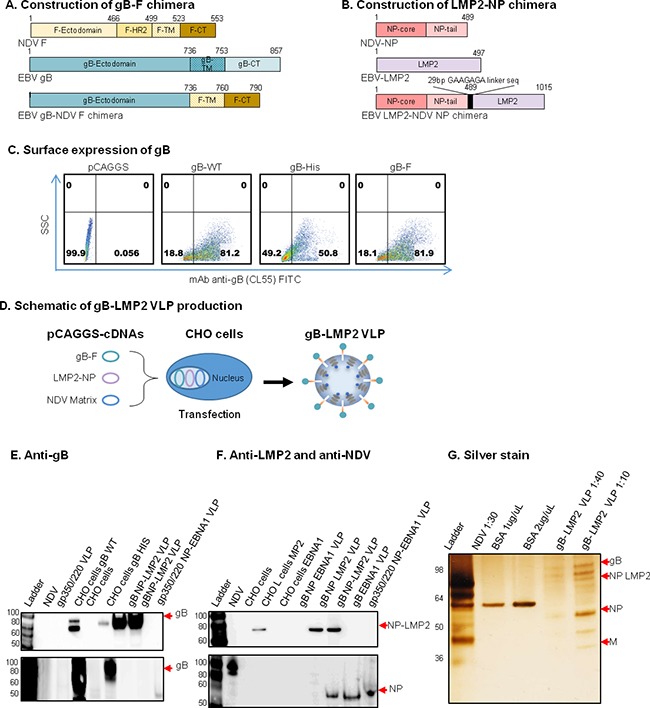
Construction, assembly, and characterization of gB-LMP2 VLPs Schematic illustrations (not to scale) of **A.** EBV gB-NDV F and **B.** EBV LMP2-NDV NP chimera plasmids. **C.** Flow cytometry for surface expression of gB on 106 CHO cells transfected with 1 μg of (from left): empty pCAGGS vector (neg. control); pCAGGS-gB-full-length (WT); His-tagged gB (pos. control); and gB-F chimera. SSC, side scatter; anti-gB mAb CL55 (known to bind gB N-terminal. **D.** pCAGGS-cDNAs of the two chimeras and NDV Matrix were co-transfected into CHO cells for VLP production as illustrated. **E-G.** Purified gB-LMP2 VLPs and transfected cells were lysed and analyzed by immunoblot to detect gB, M, NP and LMP2 proteins. (E) mAb-CL55 (N-terminal) and BA23 (C-terminal). (F) Anti-LMP2 (top), polyclonal anti-NDV (bottom panel) were used to detect respective proteins. Anti-gB: un-transfected CHO cells, and purified gp350/220 VLPs, were neg. controls; CHO cells transfected with gB-WT or gB-NDV-F chimera, gB-His-tagged were pos. controls. (G) Purity of the gB-LMP2 VLPs were analyzed by silver stain, VLP component proteins of predicted sizes were detected as indicated; NDV was pos. control, BSA was used for quantification.

Following the success of producing VLPs that express both surface glycoprotein and intracellular protein, we developed another VLP packaging EBV glycoprotein, gB, with full length LMP2. LMP2 is recognized by T cells and is immunogenic in the control of post-transplant lymphoproliferative diseases. We reasoned that it would be an excellent vaccine component when combined with gB, which was recently shown to also play an integral role in EBV entry [32]. To construct the gB-LMP2 VLP, we synthesized chimeric sequences of: gB ectodomain fused to the NDV F TM/CT domains (gB-F, Figure 3A) and full-length LMP2 fused to NDV NP (LMP2-NP, Figure 3B). The fragments were individually cloned into pCAGGS and sequence fidelity was verified. To confirm surface expression of gB, we used flow cytometry with anti-gB (CL55). CHO cells transfected with the pCAGGS-gB-F chimera expressed gB similar to cells transfected with pCAGGS-gB (full length) and His-tagged gB (positive controls), while cells transfected with empty pCAGGS vector (negative control) did not express gB (Figure 3C).

To assemble and produce the gB-LMP2 VLPs, we co-transfected equal amounts of pCAGGS-gB-F, and pCAGGS-LMP2-NP chimeras into 40 T175 flasks seeded with CHO cells, together with pCAGGS-M (Figure 3D). Supernatant was collected from transfected cells from 24-96 h post-transfection and VLPs were purified as previously described [18, 20]. To confirm the production of gB, LMP2, and NP VLP components, purified gB-LMP2 VLPs were analyzed for protein composition and molecular weight using immunoblot (Figure 3E–3F) and silver stain (Figure 3G). Anti-gB (CL55, C-terminal) detected gB protein in CHO cells transfected with pCAGGS-gB (full length), pCAGGS-gB-His (positive controls), and in purified gB-LMP2 VLPs but not in NDV lysate, untransfected CHO cells (negative controls), or in purified gp350/220 or gp350/220-EBNA1 VLPs (Figure 3E, upper. Anti-gB (BA23, N-terminal) detected gB protein in CHO cells transfected with pCAGGS-gB (full length) and pCAGGS-gB-His, but not in NDV lysate, untransfected CHO cells, or purified gB-LMP2 (confirms successful fusion of gB ectodomain-only in the chimera), gp350/220, or gp350/220-EBNA1 VLPs (Figure 3E, lower). Anti-LMP2 detected LMP2-NP in CHO cells transfected with pCAGGS-LMP2-NP (positive control) and in purified gB-LMP2 VLPs, but not in NDV lysate or untransfected CHO cells (negative controls), or purified gB-EBNA1 or gp350/220-EBNA1 VLPs (Figure 3F, upper). Anti-NDV detected NP and relevant NP chimeras, in NDV lysate (positive control) and in gp350/220, gB-LMP2, and gp350/220-eGFP VLPs, but not in untransfected CHO cells (negative control) or CHO cells transfected with pCAGGS-gB or pCAGGS-gB-His (Figure 3F, lower). Silver stain was used to detect VLP component proteins as well as to assess VLP purity; NDV lysate was used as a positive control and BSA was used for quantification of protein. Bands for gB-LMP2 VLP component proteins of predicted sizes were detected for gB (100 Kda), chimeric LMP2-NP (80 Kda), NDV NP (53 Kda), and NDV M (40 Kda) (Figure 3G).

### VLPs stimulate both IgG-specific antibody and EBNA1- and LMP2-specific T-cell responses in immunized BALB/c mice

**Figure 4:**
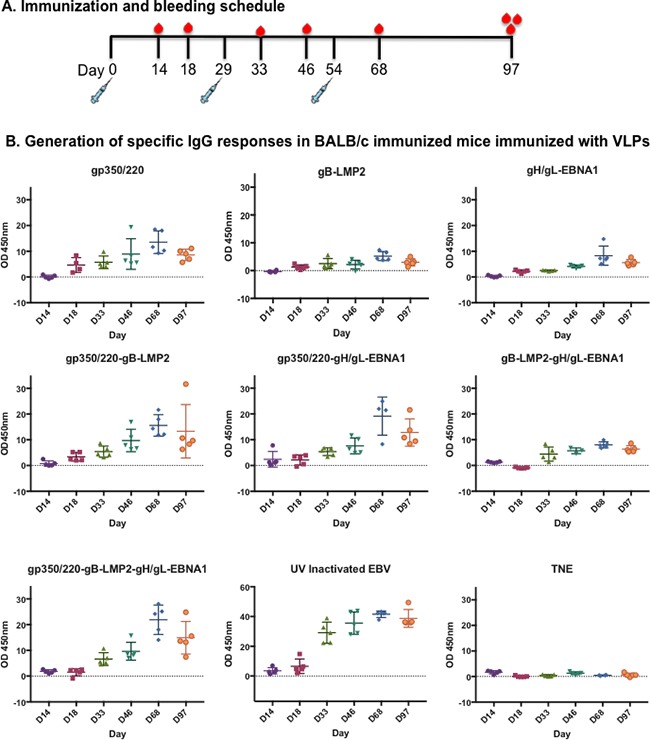
Immunization and generation of EBV-specific IgG antibodies in immunized BALB/c mice **A.** Immunization and bleeding schedule of 6–8-week-old female BALB/c mice. A total of 9 groups of BALB/c were immunized with 10 mg of purified gp350/220, gB-LMP2, gH/gL-EBNA1 VLPs or a combination of gp350/220 and gB-LMP2; gp350/220 and gH/gL-EBNA1; gB-LMP2 and gH/gL-EBNA1; or gp350/220, gH/gL-EBNA1, and gB-LMP2 VLPs. Two groups were also immunized with purified UV-inactivated EBV (10 mg) and TNE buffer, as positive and negative controls, respectively. **B.** EBV-glycoprotein IgG specific Ab titer in sera from immunized BALB/c mice was determined using lysate from lytically induced AGS-Akata cells as target, and detected by ELISA at Days 14, 18, 33, 46, 68, and 97 for each individual mouse. ELISA assay was repeated at least three times. Results are expressed as mean ± standard deviations (SD).

To test the efficacy of individual EB VLPs to generate antibodies and induce T-cell responses, nine groups (n=5/treatment) of 6–8-week-old female BALB/c mice were immunized intraperitoneally three times (Day 0, 29, 54) with 10 μg of purified gH/gL-EBNA1 VLPs, gB-LMP2 VLPs, or gp350/220 VLPs in 0.5ml of TNE buffer (100 mM Tris; 2.0 M NaCl; 10 mM EDTA; ~10% sucrose; pH 7.4). Purified UV-inactivated EBV (10 μg) was used to immunize mice as positive control; TNE buffer served as negative control. To test whether there are additive or synergistic effects of using two or more immunogens in the vaccine, we immunized groups of 5 mice with two or three individual VLPs (10 μg each of: gH/gL-EBNA1 and gB-LMP2; gH/gL-EBNA1 and gp350/220; gB-LMP2 and gp350/220; or gH/gL-EBNA1, gB-LMP2, and gp350/220). Mice were tail vein-bled to obtain serum at two and four weeks after primary immunization and again once after both secondary (Day 29) and tertiary (Day 54) boost immunizations (Figure 4A). Mice were sacrificed and terminal bleed was collected on Day 97.

To demonstrate the ability of VLPs to induce EBV subunit-specific antibody responses, we used enzyme linked immunosorbent assay (ELISA) to assess antibody titers against gH/gL, gB, gp350/220, and UV-inactivated EBV (positive control) in collected sera. First, lysates from lytically induced AGS-EBV-eGFP cells, which express all viral glycoproteins, were used as binding targets. Sera collected at Days 14, 18, 33, 46, 68, and 97 (terminal bleed) were used to determine the presence of IgG-specific antibodies (Figure 4B). All VLP-immunized mice generated EBV-glycoprotein-specific IgG antibody responses that significantly increased following primary and secondary boost immunizations, compared to TNE (negative control). The increase in EBV-specific antibody response peaked at Day 68 for all VLP-immunized mice, similar to UV-inactivated EBV-immunized mice (positive control). Similar slope patterns were observed in the mice immunized with all VLPs (gp350/220, gB-LMP2, and gH/gL-EBNA1) compared to UV-inactivated EBV. gp350/220 VLP-immunized mice generated the highest amount of IgG-specific antibodies, compared to mice immunized with gB-LMP2 VLPs or gH/gL-EBNA1 VLPs. To reduce non-specific background and to compare specific EBV glycoprotein gp350/220 IgG antibody titers in the sera of mice immunized with UV-inactivated EBV, we used gp350 soluble proteins as binding targets for ELISA (Supplementary Figure S2). There are no commercially available purified gB or gH/gL proteins to allow us characterize antibody specificity to gB and gH/gL. Individual EBV glycoprotein-specific antibodies were detected in all immunized mice, and again UV-inactivated EBV generated the highest titer of IgG-specific antibodies, whether we used transfected ELL-0 cells or soluble gp350 purified from HEK-293 cells (Immune Tech).

**Figure 5:**
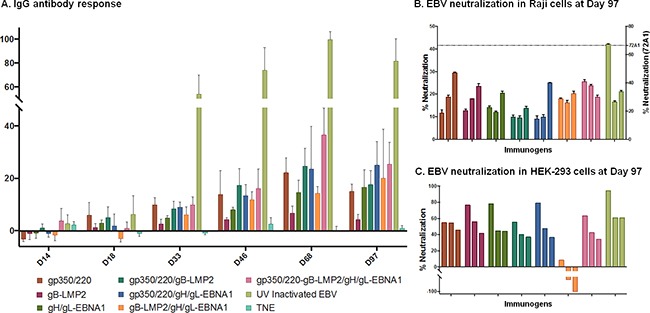
Antibody titer and percent EBV neutralization **A.** The absorbance resulting from serum antibody binding to plates coated with lysate from lytically induced AGS infected with EBV-Akata strain is shown for each vaccination group for individual mice. Absorbance data are shown as the mean ± the SEM for five mice from three independent ELISAs. Data points represent individual mice. **B-C.** Pooled sera from terminal bleed (Day 97) from 5 animals/immunization treatment were pre-incubated with eGFP-EBV in 1:2.5, 1:5 or 1:10 dilution) as shown in the bar graphs for each immunogen, then incubated at 37°C for 48 h with Raji cells (B) or HEK-293 cells (C) seeded in total volume of 100μl of serum free media; EBV-EGFP+ cells were enumerated by flow cytometry. Neutralizing anti-gp350/220 mAb (72A1; dashed line) served as positive control and neutralized >70% of the EBV infection in Raji cells and 100% in HEK 293 cells (data not shown). TNE served as negative control and was used to normalize percent infection. The neutralization assays were repeated three times in Raji cells and twice in HEK-293 cells. Results are expressed as mean ± standard deviation (SD) for Raji cells. Horizontal black lines terminating in short vertical lines compare sets of neutralization experiments with 2-sided p-values <0.05.

To assess the ability of VLP-immunized sera to neutralize infection *in vitro*, we quantitated sera collected at Days 14, 18, 33, 46, 68, and 97. (Figure 5A) Infection of HEK-293 or Raji cells with purified AGS-EBV-eGFP was used to conduct neutralization using pooled sera from Day 97 sera. HEK-293 and Raji cell lines are susceptible to EBV infection, and represent both epithelial and B cell lineages. The virus titer was determined as described [20]. The percent of eGFP^+^ cells was determined by flow cytometry as previously described [20]. Purified antibody known to block EBV infection (anti-gp350/220 [72A1]) [55, 56] served as a positive control (data not shown). Effective neutralization titer was defined as 50% inhibition of infection, compared to control serum samples from EBV-seronegative animals.

When 5μl of AGS-EBV-eGFP virus was pre-incubated with a serial dilution (1:2.5, 1:5, and 1:10) of sera from TNE-immunized mice (negative control), fluorescence dropped from 19.1% (virus alone) to 10% in HEK-293 cell line. Similar results were observed in Raji cells. In contrast, when AGS-EBV-eGFP was pre-incubated with serially diluted sera from mice immunized with gH/gL-EBNA1 VLPs, gB-LMP2 VLPs, gp350/220 VLPs, or UV-inactivated EBV, infection was reduced in a dose-dependent manner compared to TNE-immunized sera (Figure 5B–5C). Purified anti-gp350/220 mAb 72A1 (5 μg/ml) (positive control) was the most effective in neutralizing EBV infection, reducing infection by >70% in both Raji and HEK-293 cells. In our hands, at 1:2.5 dilution, sera from mice immunized with UV-inactivated EBV significantly neutralized EBV infection of Raji cells compared to sera from mice immunized with gp350/220 (p = <0.0001), gB-LMP2 (p = <0.0001) or gH/gL-EBNA1 (p = <0.0001) VLPs (Figure 5B). Between the VLPs, sera from mice immunized with gH/gL-EBNA1 VLPs significantly reduced EBV infection of Raji cells compared to sera from mice immunized with gp350/220 (p = 0.0374) or gB-LMP2 (p = 0.0112) VLPs. There was no significant difference in virus neutralization between sera from mice immunized with gp350/220 and gB-LMP2 VLPs. Similar trends were observed at 1:5 dilution and at the highest dilution (1:10). At 1:10 dilution, sera from UV-inactivated EBV mice was superior to gp350/220 (p = 0.0021) VLPs. However, no significant difference was observed between UV-EBV and either gH/gL-EBNA1 or gB-LMP2 VLP at this dilution. Between the VLPs, similar to the 1:2.5 dilution, serum diluted 1:10 from gH/gL-EBNA1 was superior to gp350/220 (p = 0.0029), but not gB-LMP2. However, at this dilution, sera from mice immunized with gB-LMP2 VLPs neutralized better than sera from gp350/220 VLPs (p = 0.026).

When we performed the neutralization assay in HEK-293 epithelial cells, sera from mice immunized with UV-inactivated EBV at the 1:2.5 dilution effectively neutralized 94.3% of EBV infection, followed by sera from mice immunized with gH/gL-EBNA1 VLPs (77.9%), gB-LMP2 VLPs (76.8%), and gp350/220 VLPs (54.9%) (Figure 5C). Overall, our experiments confirmed that sera from mice immunized with gH/gL-EBNA1 or gB-LMP2 VLPs neutralized EBV infection *in vitro* better than gp350/220 VLPs in both Raji and HEK-293 cells.

Surprisingly, sera from mice immunized with a combination of two VLPs (gH/gL-EBNA1 and gB-LMP2, gH/gL-EBNA1 and gp350/220, or gB-LMP2 and gp350/220), or with all three VLPs, did not significantly reduce EBV infection compared to the single glycoprotein immunogens, in either of the cell lines used. In fact, pre-incubation of EBV with sera from mice immunized with both gB-LMP2 and gH/gL EBNA1 somewhat increased infection of HEK-293 cells.

**Figure 6:**
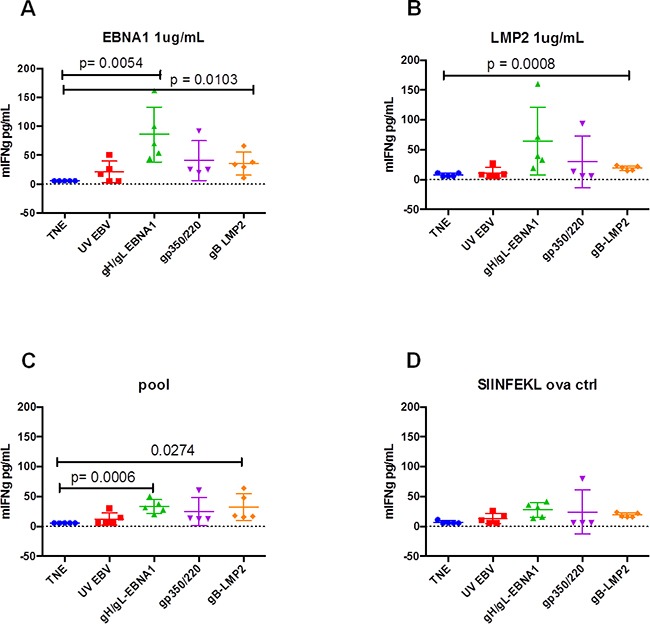
*In vitro* activation of T cells isolated from BALB/c mice immunized with gH/gL-EBNA1, gB-LMP2, or gp350/220 VLPs A group of five BALB/c mice per treatment were immunized with 10 μg of gH/gL-EBNA1, gp350/220, and gB-LMP2 VLPs and boosted twice at Days 29 and 54 without adjuvants. UV-inactivated EBV and TNE served as positive and negative controls, respectively. At day 97 mice were sacrificed, red blood cells lysed and 5×10^5^ splenocytes from sacrificed mice were assessed for their response to *in vitro* stimulation with 1 μg/mL of synthetic peptides derived from EBNA1_(HPVGEADYFEY)_, LMP2_(CLGGLLTMV)_, or Promix EBV peptide pool. After overnight culturing, the supernatants were tested for IFN-γ release by ELISA. Splenocytes from mice immunized with gH/gL-EBNA1 VLPs or gB-LMP2 VLPs generated significantly higher IFN-γ than mice immunized with UV-inactivated EBV and gp350/220 VLPs. SIINFEKL ovalbumin was used as a negative control. The experiment was replicated three times. Results are expressed as mean ± standard deviations (SD).

To quantitate T-cell activation in immunized mice, we stimulated splenocytes (5×10^5^) from sacrificed mice *in vitro* with 1 μg/mL synthetic peptide antigens derived from EBNA1_(HPVGEADYFEY),_ LMP2_(CLGGLLTMV)_, or Promix EBV-peptide pool (PeproTech). After overnight culturing, we used ELISA to test supernatants for release of IFN-γ, an indicator of T-cell activation. Splenocytes from mice immunized with gH/gL-EBNA1 VLPs or gB-LMP2 VLPs generated significantly higher IFN-γ in response to EBNA1 (Figure 6A) or LMP2 (Figure 6B) peptides than mice immunized with UV-inactivated EBV or gp350/220 VLPs, relative to mice immunized with TNE buffer alone (negative control). Stimulation with a pool of EBV peptides gave similar results (Figure 6C). SIINFEKL ovalbumin was used as a negative control (Figure 6D) and concavalin A and IL1β were used as model antigens (data not shown). These results confirm that gH/gL-EBNA1 and gB-LMP2 VLPs can stimulate EBV-specific T-cell-mediated immune response *in vivo.*

## DISCUSSION

We have developed a novel platform for generating polyvalent EB VLPs that are produced in CHO cells and incorporate and package both EBV surface glycoproteins and intracellular proteins. Combined expression of surface glycoprotein(s) known to mediate viral entry (gp350/220, gH/gL, or gB), and proteins important for establishment of viral latency (EBNA1 or LMP2), allowed us to generate unique VLPs that can serve as polyvalent prophylactic and therapeutic EBV vaccine candidates. To our knowledge, the incorporation of select EBV surface glycoproteins and packaging of viral T-cell antigens inside the VLPs is unique to our study; we consider it a critical advancement in the development of a safe and effective polyvalent EBV vaccine, compared to previously reported candidates [13, 20, 42]. Importantly, our vaccine candidates induced nAbs and also generated EBNA1- or LMP2-specific T cells in immunized wild-type BALB/c mice. Thus, we have established that generating a polyvalent prophylactic and therapeutic vaccine against EBV is feasible.

Because of gp350/220's critical role as the attachment protein in viral entry, and observations that mAb directed against the protein (72A1) blocks EBV infection *in vitro* [56], to date all efforts to develop prophylactic EBV vaccines have primarily focused on this protein, with variable success [2, 11, 52]. However, our data shows pooled serum from mice immunized three times with gH/gL-EBNA1, without any adjuvant, neutralized EBV infection more effectively than pooled sera from mice immunized with gp350/220 VLPs, in both B cells and epithelial cells. The importance of these alternate glycoproteins is supported by other evidence, including that recombinant EBV lacking gH does not infect either epithelial or primary B cells [57]. Although antibodies to gH/gL are not robustly produced *in vivo* during natural infection (perhaps due to masking by the immunodominant gp350/220), immunization of animals with recombinant gH or gH/gL can boost immunogenicity and generate antibodies capable of blocking EBV infection [28, 53, 58, 59]. The ability of gH/gL antibodies to neutralize infection is also well-conserved in herpes simplex virus-1 [60, 61], cytomegalovirus [62], and Kaposi sarcoma herpesvirus [63]. Furthermore, monoclonal antibodies to the gH protein or the gH/gL complex block EBV infection, supporting a critical role for gH/gL in EBV infection [30, 64].

Importantly, a recent report that rabbits immunized with 25 μg of gH/gL or gB, and enhanced with aluminum hydroxide as an adjuvant, generated superior nAb responses (100-fold and 20-fold, respectively) to gp350/220 supports our findings [53]. Although no specific nAbs to EBV gL or gB have been reported in patients to date [11], gB-specific nAbs have been tested in several clinical trials to prevent neonatal cytomegalovirus infection [65–67]. In our study, pooled sera from mice immunized with UV-inactivated EBV still had the best neutralization effect in epithelial and B-cell lines, which suggests that an effective EBV prophylactic vaccine may require inclusion of more than two glycoproteins, since the EBV entry mechanism involves multiple surface glycoproteins.

Surprisingly, sera from mice immunized with a mixture of both gH/gL-EBNA1 and gB-LMP2 VLPs had a contrasting outcome: supporting rather than blocking infection of epithelial cells. Whether this is true in natural infection, or whether co-expression of the three glycoproteins together on the surface of a single VLP (since gB, gH, and gL form a heteromeric complex) will have a different outcome remains to be explored. Pooled sera from mice immunized with both gp350/220 and gB-LMP2 VLPs or with gB-LMP2, gp350/220, and gH/gL-EBNA1 VLPs did not provide a better neutralization outcome than the use of single immunogens in both cell lines tested. Only pooled sera from mice immunized with both gp350/220 and gH/gL-EBNA1 VLPs achieved an additive effect in the HEK-293 cell line, suggesting that a strategy combining these two immunogens might achieve sterile immunity, particularly if it is enhanced by the use of adjuvants, as for the case of three FDA-approved human papillomavirus vaccines.

In summary, our study demonstrates that EB VLPs can be stably produced in CHO cells, which grow to high density in suspension [68]. CHO cells can also be grown without FBS, allowing for more efficient and cost-effective preparation, and setting the stage for large-scale production. This is yet another benefit to the production of VLPs in the CHO cell line, an FDA-approved vehicle for production of almost all currently licensed biologics, and is a significant step toward producing a safe EBV vaccine. The appearance of adventitious viral products, a typical artifact of production in cell lines of human origin, and a major concern to regulatory authorities, has not been reported for CHO cells [69]. These VLPs were found to be immungenic by generating nAbs and EBV-specific T-cell responses in immunized mice, suggesting they are a potentially safe and effective EBV polyvalent vaccine.

In future studies, we aim to improve our current vaccines by generating a polyvalent vaccine candidate that combines gp350/220-gH/gL-EBNA1-LMP2 as a single VLP. It might also be important to consider including gp42 as part of the proposed pentameric VLPs, as nAbs to gp42 have been detected in human sera [54], and interaction between gp42/gH/gL is known to mediate viral infection in B cells [34]. We will also explore whether use of adjuvants will improve immunogenicity to achieve optimal neutralization. Testing these vaccines in recently developed humanized mouse-disease models (e.g., Burkitt lymphoma, PTLD) [70–73] will help inform whether the candidate vaccine can serve as an alternative to adoptive immunotherapy or as a cancer immunotherapy agent against EBV-associated cancers.

## MATERIALS AND METHODS

### Virus and Cell lines

The B95-8 cell line (marmoset B cell line) harboring recombinant Epstein-Barr virus (EBV) expressing an enhanced green fluorescence protein (eGFP) reporter at the LMP2A locus (B95-8-eGFP) [74] was a generous gift of Dr. R. Longnecker (Northwestern University, Chicago). The AGS cell line (human gastric adenocarcinoma cell line) infected with a recombinant Akata EBV expressing eGFP at the thymidine kinase locus (AGS-EBV-eGFP) [75, 76] was a generous gift of Dr. L. Selin (University of Massachusetts Medical School).

HEK-293 (human embryonic kidney), Raji (EBV-positive Burkitt lymphoma), CHO, ELL-0 (chicken embryo), and HB168 (72A1 murine hybridoma) cell lines were all purchased from the American Type Culture Collection. B95-8-eGFP, Raji, and HB168 cells were cultured in RPMI media. HEK-293, CHO, and ELL-0 cells were cultured in Dulbecco's Modified Eagle's medium (DMEM). AGS-EBV-eGFP cells were cultured in Ham's F-12 media. Both B95-8-eGFP and AGS-EBV-eGFP media were supplemented with 50μg/ml of neomycin (G418). All media contained 1% L-glutamine, 10% heat-inactivated fetal bovine serum (FBS), and 2% penicillin-streptomycin unless otherwise specified. Lytic induction and purification of virus from B95-8-eGFP and AGS-EBV-eGFP cell lines harboring EBV-eGFP was performed as described [77]. Purified B95-8-eGFP and AGS-EBV-eGFP viruses were titered in both HEK-293 and Raji and HEK-293 cells and stocks kept in -80^°^C for subsequent experiments.

### Antibodies

Primary antibodies: Mouse monoclonal 72A1 (anti-gp350/220) was purified from HB168, a producer cell line, as described [56]. Rabbit polyclonal anti-Newcastle disease virus (NDV) has been described [78] and was a gift of Dr. T. Morrison, University of Massachusetts Medical School, Worcester, MA. Rat monoclonal anti-EBV nuclear antigen 1 (EBNA1; IH4-1) was a gift of Dr. F. Grässer, Institut für Virologie, Germany [79]. Goat polyclonal anti-EBNA1 (Gly-Arg regions) and rat monoclonal anti-EBV latent membrane protein 2 (LMP2A; 14B7) were purchased from Abcam. Mouse monoclonal anti-eGFP (GSN149) was purchased from Sigma. Anti-gH/gL (E1D1, CL40) and anti-gB (CL55, N-terminal; BA23, C-terminal) were gifts of Dr. L. Hutt-Fletcher (Louisiana State University Health Sciences Center, Shreveport, LA); mouse monoclonal E1D1 recognizes the gH/gL complex and partially blocks integrin binding, likely on the domain I/domain II interface [80]; mouse monoclonal CL40 recognizes the gH/gL complex; mouse monoclonal CL55 recognizes the gB ectodomain; rabbit polyclonal BA23 recognizes the gB C-terminal domain.

Secondary antibodies: Horseradish peroxidase (HRP)-conjugated goat anti-mouse IgG (total and isotype specific), goat anti-rabbit, goat anti-rat, or rabbit anti-goat antibodies for immunoblot or ELISA were purchased from Bio-Rad. Alexa Fluor (AF) 488- or AF594-conjugated goat F(ab’)2anti-mouse IgG (H+L) for flow cytometric analyses or confocal microscopy were purchased from Life Sciences Tech.

### Plasmid vectors

Construction of pCI-puro has been previously described [26]. All other cDNA sequences were synthesized by Genewiz, South Plainfield, NJ, cloned into the pCAGGS mammalian expression vector [81], and fidelity was verified by sequencing the cDNAs. All cDNA sequences encoding EBV surface glycoproteins and latent membrane proteins were derived from B95-8 virus [82]. VLP component plasmids pCAGGS-fusion (F), pCAGGS-hemagglutinin-neuraminidase (HN), pCAGGS-matrix (M), and pCAGGS-nucleocapsid protein (NP) derived from NDV (Avulavirus) have been described [83].

#### pCAGGS-gp350/220-F

Full-length EBV gp350/220 (BLLF1) from the BamHI L fragment of EBV genome strain B95-8 [82] was amplified by PCR as previously described [20]. To construct the gp350/220-F chimera, the gp350/220 ectodomain (amino acids (aa) 1-864) was fused to the NDV F transmembrane (TM) and cytoplasmic (CT) domains (aa 499-553).

#### pCAGGS-gH-F, pCAGGS-gL-HN, and pCAGGS-gB-F

The rationale for fusion of type 1 membrane protein to NDV F protein and type 2 membrane protein to NDV HN has been described [19]. Chimeras were constructed as follows: gH-F chimera, NDV F TM/CT domains (aa 499-553) were fused to the ectodomain (aa 1-679) of EBV gH (gp85; type 1); gL-HN chimera, NDV HN TM/CT domains (aa 1-45) were fused to the ectodomain (aa 25-137) of EBV gL (gp25; type 2); gB-F chimera, NDV F TM/CT domains (aa 499-553) were fused to the ectodomain (aa 1-736) of EBV gB (gp110; type 1).

#### pCAGGS-eGFP-NP, pCAGGS-EBNA1-NP, and pCAGGS-LMP2-NP

Full-length NDV-NP (aa 1-489) was synthesized and fused to a 29-aa (PATLSLPPDHPNSSARSPPP DPGAAGAGA) linker sequence [84]. To construct the chimeras, NDV NP-GAAGAGA was fused to the following components: eGFP-NP chimera, eGFP (amino acids 1-241); EBNA1-NP chimera, truncated EBV EBNA1 (aa 326-641; the truncated sequence lacks the Gly-Ala regions known to impair presentation of *cis*-linked sequences to MHC class I); LMP2-NP chimera, full-length EBV LMP2 (aa 1-497).

#### Control plasmids

cDNA sequences of genes encoding full-length: gp350/220 (aa 1-907), gH (aa 1-707), gL (aa 1-137), gB (aa 1-790), gB-His (aa 1-742), eGFP (aa 1-241), EBNA1 (aa 1-641), and LMP2 (aa 1-497 were individually cloned into pCAGGS for use as experimental controls.

### Transfection

1.0 μg/well of empty pCAGGS, and pCAGGS vectors containing full-length gp350/220, gp350/220-F, full-length gH, gH-F, full-length gL, gL-HN, full-length gB, gB-F, full-length eGFP, eGFP-NP, full-length EBNA1, EBNA1-NP, full-length LMP2, and LMP2-NP were individually transfected into 80% subconfluent CHO cells seeded in six-well tissue culture plates using Lipofectamine and Reagent Plus (Life Sciences Technologies) following manufacturer's recommendations. To assess formation of the gH/gL complex, pCAGGS-gH-F and pCAGGS-gL-HN or pCAGGS-gH and pCAGGS-gL plasmids were co-transfected in combination into CHO cells.

### VLP assembly and production

For VLP preparation, equal amounts (8 μg/plasmid) of the relevant plasmids were co-transfected into 80% confluent CHO cells seeded in T-175cm^2^ flasks; supernatant from transfected cells was collected and VLPs were purified as previously described [18, 20]. VLPs were constructed using co-transfection of relevant pCAGGS plasmids as follows: gp350/220-F VLP – gp350/220-F, NDV-NP, and M; gp350/220-eGFP VLP – gp350/220-F, eGFP-NP, M, and pCIpuro; gp350/220-EBNA1 VLP – gp350/220-F, EBNA1-NP, and M; gH/gL-EBNA1 VLP – gH-F, gL-HN, EBNA1-NP, and M; and gB-LMP2 VLP – gB-F, LMP2-NP, and M.

### Imaging eGFP-positive cells

Cells transfected with plasmids expressing eGFP (pCAGGS-eGFP, pCAGGS-eGFP-NP) or untransfected CHO cells (negative control) were imaged using filters set for FITC, with an excitation wavelength of 488 nm resulting in emission at 507 nm, as described [77].

### Generation of stable CHO cells producing gp350/220-eGFP VLPs

To generate a stable CHO cell line producing gp350/220-eGFP VLPs, 1.0 μg/well of pCAGGS-gp350/220-F, pCAGGS-eGFP-NP, pCAGGS-M, and pCIpuro plasmids were co-transfected into 1×10^5^ CHO cells seeded in a six-well tissue culture plate. At 48 hours post-transfection, 10 μg/ml of puromycin was used to select transfected cells. Individual colonies were cloned and expanded, followed by flow cytometric sorting using anti-gp350/220 antibody (mAb 72A1) and eGFP. Cells were seeded and passaged 10 times and VLP production was assessed in each passage by immunoblot as described below.

### Flow cytometry, silver stain, and immunoblot detection of proteins

To assess surface expression of relevant protein(s) and protein complexes by flow cytometry, transfected cells were harvested at 48 hours post-transfection and stained with relevant primary and secondary antibodies. Flow cytometric analysis was performed on an LSRII benchtop FC (Becton-Dickinson, B-D) and data was analyzed using CellQuest Pro Version 4.0.1 (BD Biosciences) and/or FlowJo Cytometry Analysis software (FlowJo, LLC) as described [20]. All the experiments were independently repeated at least three times unless otherwise specified.

To assess expression of relevant protein(s) and protein complexes using silver stain and immunoblot, purified VLPs, NDV, and B95-8-eGFP, untransfected CHO cells (negative control), or cells transfected with various pCAGGS plasmids were lysed in RIPA buffer (Boston Bioproducts) containing complete protease inhibitor cocktail (Roche Applied Science). Lysates were incubated on ice for 30 min, and then centrifuged for 5 min at 18,407 rcf in a microcentrifuge. The protein content of the lysates was determined by Bradford assay using Coomassie Brilliant Blue (Sigma). Lysates were boiled for 5 min in Laemmli SDS sample buffer (Boston Bioproducts) under non-reducing or reducing conditions. A known quantity of protein lysate was separated on a 4–12% gradient polyacrylamide gel. Pierce silver stain kit (Thermo-Fisher) was used to detect proteins following manufacturer's recommendations. All the experiments were independently repeated at least three times unless otherwise specified. Immunoblot analyses were performed by transferring proteins from the gel to a PVDF membrane using iblot (Thermo-Fisher). Membranes were pre-incubated with 5% non-fat dry milk (LabScientific) for 30 min and detected with relevant primary and secondary antibodies as previously described [26, 85].

### Immunization of BALB/c mice

Nine groups (n=5/treatment) of 8–10-week-old female BALB/c mice were immunized intraperitoneally three times (Day 0, 29, 54) with 10 μg of purified gp350/220-F, gH/gL-EBNA1, or gB-LMP2 VLPs, or with a combination of 10 μg of each of two or three of the VLPs, in 0.5 ml of TNE buffer (100 mM Tris; 2.0 M NaCl; 10 mM EDTA; ~10% sucrose; pH 7.4). Purified UV-inactivated EBV (B95-8-eGFP) and gp350/220-F VLPs served as positive controls; TNE buffer alone served as negative control. To assess the short, mid, and long-term immunogenic nAb responses, mice were tail vein-bled to obtain serum at two and four weeks after primary immunization and again once after both secondary (Day 29) and tertiary (Day 54) boost immunizations. On Day 97, mice were sacrificed and terminal bleed (~1 mL) was collected for use in determination of antibody titer and EBV neutralization assays. Splenocytes were collected from sacrificed mice for determination of EBNA1- and LMP2-specific T-cell responses as described [86].

### Determination of antibody titer by ELISA

To assess the EBV IgG-specific antibody titer for each immunized mouse, enzyme linked immunosorbent assay (ELISA) was performed as previously described [87] using various target antigens. Target antigens comprised: lysates from induced AGS-EBV-eGFP cells; lysates from ELL-0 cells transfected with pCAGGS plasmids containing full-length gp350/220, or soluble gp350 ectodomain (aa 4-863) proteins (Immune Technology Corp). Briefly, 4 μg of pCAGGS plasmids expressing relevant surface glycoproteins were transfected into ELL-0 cells seeded in T-75 cm^2^ flasks. Triton X lysis buffer was used to extract lysates from induced AGS-EBV-eGFP cells or transfected ELL-0 cells (48 hours post-transfection). Dilutions of the cell extracts were adjusted so that the amount of glycoprotein targets was comparable from experiment to experiment, as determined by Western blotting and Bradford assay. Untransfected ELL-0 or uninduced AGS-EBV-eGFP cell extracts served as negative controls. ELL-0 cells provided less non-specific background in the ELISA assay.

To perform ELISA, 96-well microtiter plates (Nunc-Immuno Plate with Maxisorp surface treatment) were coated with 50 μl/well of lysates expressing relevant viral glycoproteins (1 μg/ml) in a phosphate-buffered saline (pH 7.2) overnight at 4°C. After blocking the plates with 2% bovine serum albumin (BSA), serial dilutions (in PBS) of sera from Days 14, 18, 33, 46, 68, and 97 (terminal bleed) were bound for 2 hours at room temperature. Antibody binding was detected using HRP-conjugated goat anti-mouse IgG secondary antibody at room temperature for 1 hour. The plates were washed five times with PBS and the chromogenic substrate 2,2′-azino-bis(3-ethylbenzothiazoline-6-sulphonic acid) (ABTS, Life Science Technologies) was added. The reaction was stopped using ABTS Peroxidase Stop Solution containing 5% sodium dodecyl sulfate (SDS) in water. To determine the antibody titer, optical density was read at 405nm (OD_405_) with ELISA reader (Spectramax® Plus 384, Molecular Devices). The highest dilution yielding an OD_405_ two times higher than that of TNE-treated mice without dilution was designated as the antibody endpoint titer. Anti-gp350/220 (mAb 72A1) was used as a standard for the ELISA assays. Three independent replicates of the experiments were performed at least three times.

### EBV neutralization assays

Pooled sera from Day 97 (terminal bleed) of mice immunized with gp350/220-F, gH/gL-EBNA1, or gB-LMP2 VLPs, or with a combination of 10 μg of each of two or three of the VLPs, UV-inactivated EBV, or TNE buffer alone were complement heat-inactivated at 56°C and diluted in RPMI without FBS. Sera were diluted by 1:2.5, 1:5, and 1:10 and incubated with 5μl of purified AGS-EBV-eGFP (titer calculated to infect at least 20% of HEK-293 cells seeded in 100μl of serum-free DMEM) for 1 hour at room temperature. To represent EBV infection of B and epithelial cells, AGS-EBV-eGFP-incubated with sera from immunized mice were used to infect 1×10^5^ Raji or 5×10^4^ HEK-293 cells, respectively, seeded in a 48-well plate. Anti-gp350/220 (72A1 nAb), and serum from TNE only-treated mice served as positive and negative controls, respectively. Plates were incubated at 37°C and the number of GFP^+^ cells was determined by flow cytometry 48 hours post-infection. All dilutions were performed in triplicate and the assays repeated at least two times for Raji cells. Due to sera limitation, neutralization in HEK-293 cells was performed only twice, that is why statistical analysis was performed in Raji cells.

### Determination of EBNA1- and LMP2-specific T-cell responses in BALB/c immunized mice

To quantitate T-cell activation in immunized mice, splenocytes (5×10^5^) from sacrificed immunized mice were stimulated *in vitro* with 1 μg/mL synthetic peptides derived from EBNA1_(HPVGEADYFEY)_, LMP2_(CLGGLLTMV)_, or Promix EBV-peptide pool (PeproTech). After overnight culturing at 37°C, supernatants were tested for IFN-γ release, an indicator of T-cell activation, by ELISA, as described above. SIINFEKL ovalbumin was used as a negative control and concavalin A and IL1β were used as model antigens [88]. All experiments were replicated three times.

### Statistical analysis

Graph Pad Prism 6 Software was used for statistical analyses of data. The differences between the nAb titers or EBNA1- and LMP2-specific T-cell responses of the immunized and non-immunized group (TNE-treated) of mice were analyzed using unpaired two-tailed t-tests for independent groups. Statistical significance of the tests was based on a p-value equal to or lower than 0.05.

## SUPPLEMENTARY FIGURES


