# Supplementary material for: Novel Epstein-Barr virus-like particles incorporating gH/gL-EBNA1 or gB-LMP2 induce high neutralizing antibody titers and EBV-specific T-cell responses in immunized mice
Source: Oncotarget. 2016 Dec 1;8(12):19255–73. doi: 10.18632/oncotarget.13770 (PMC5386682; doi:10.18632/oncotarget.13770)
Supplement: Supplementary file 1 [file oncotarget-08-19255-s001.pdf]

Novel Epstein-Barr virus-like particles incorporating gH/gL-EBNA1 or gB-LMP2 induce high neutralizing antibodies and EBV-specific T-cell responses in immunized mice

SUPPLEMENTARY FIGURES

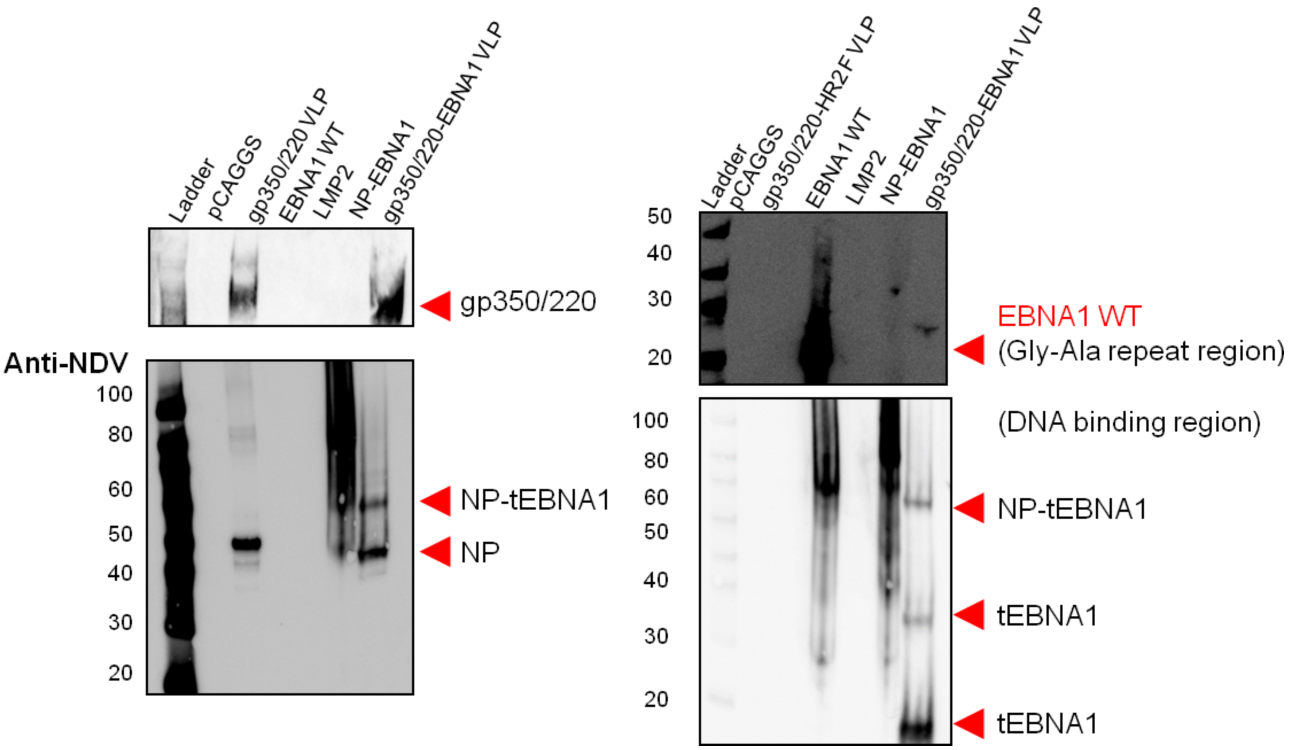

Supplementary Figure S1: Characterization of gp350/220-EBNA1 VLPs. Supernatants containing independent VLP preparations were purified, lysed, and analyzed by immunoblot with Abs against gp350/220, EBNA1, and Newcastle disease virus (NDV), as indicated. CHO cells transfected with independent plasmids were also lysed and served as controls. As expected, gp350/220, NP, and EBNA1 were detected as components of purified gp350/220 and gp350/220-EBNA1 VLPs.

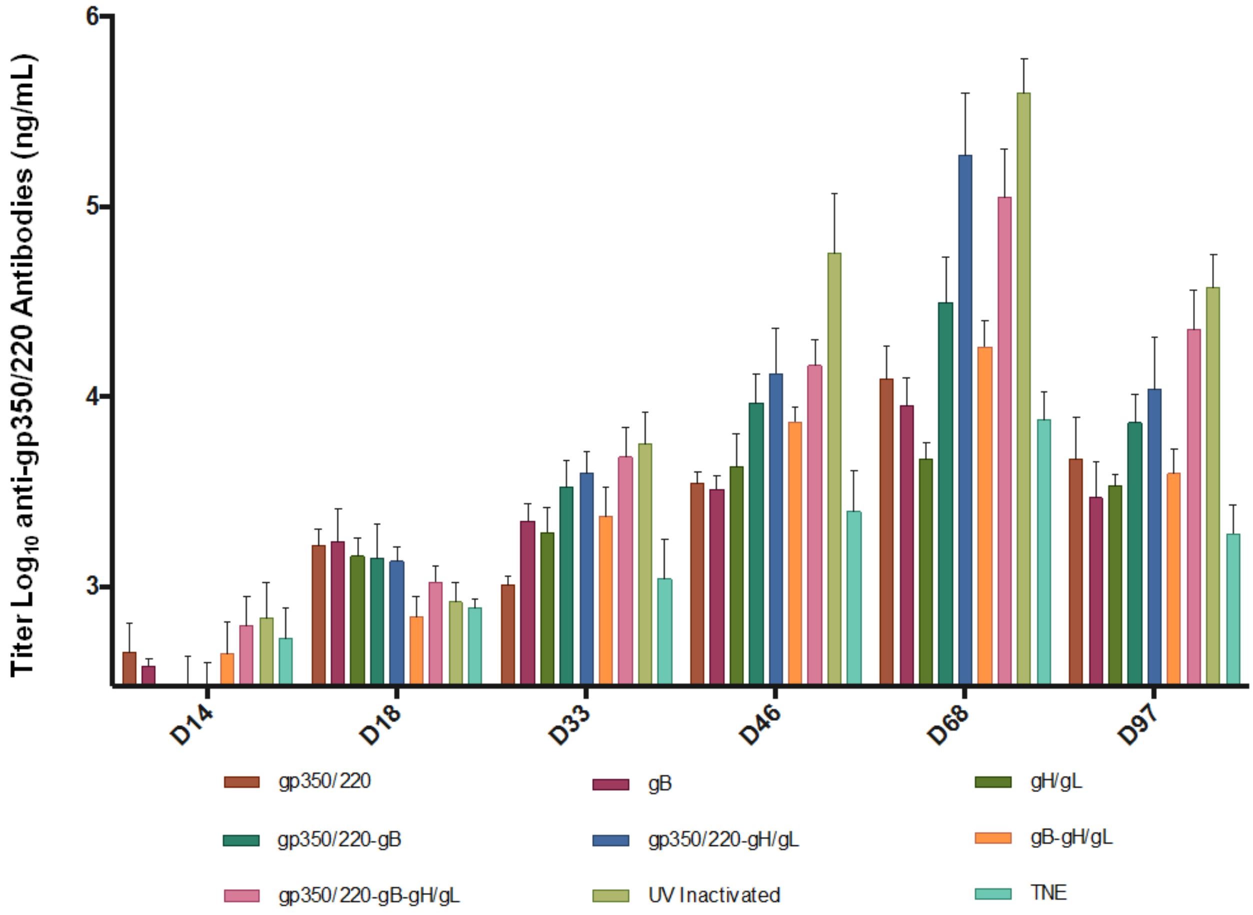

Supplementary Figure S2: Immunization and generation of EBV-specific IgG antibodies in immunized BALB/c mice. EBV-glycoprotein IgG-specific Ab titer in sera from immunized BALB/c mice was determined using purified gp350/220 as a target, and detected by ELISA at Days 14, 18, 33, 46, 68, and 97 for each individual mouse. The experiment was repeated at least three times.
